# The intricate role of annexin A2 in kidney: a comprehensive review

**DOI:** 10.1080/0886022X.2023.2273427

**Published:** 2023-11-13

**Authors:** Juan Lei, Pingping Sun, Jingyi Sheng, Hongri Wang, Yifan Xie, Jiayu Song

**Affiliations:** a Department of Pediatric Nephrology, The Second Affiliated Hospital of Nanjing Medical University, Nanjing, Jiangsu, P.R. China; b Department of Internal Medicine, Beijing Chao-Yang Hospital, Capital Medical University, Beijing, P.R. China; c Department of Rheumatism and Immunology, Children’s Hospital of Nanjing Medical University, Nanjing, Jiangsu, P.R. China

**Keywords:** Annexin A2, Ca^2+^-dependent, acute kidney injury, chronic kidney disease, renal cell carcinoma, nephrolithiasis

## Abstract

Annexin A2 (Anxa2) is a calcium (Ca^2+^)-regulated phospholipid binding protein composed of a variable N-terminus and a conserved core domain. This protein has been widely found in many tissues and fluids, including tubule cells, glomerular epithelial cells, renal vessels, and urine. In acute kidney injury, the expression level of this protein is markedly elevated in response to acute stress. Moreover, Anxa2 is a novel biomarker and potential therapeutic target with prognostic value in chronic kidney disease. In addition, Anxa2 is associated not only with clear-cell renal cell carcinoma differentiation but also the formation of calcium-related nephrolithiasis. In this review, we discuss the characteristics and functions of Anxa2 and focus on recent reports on the role of Anxa2 in the kidney, which may be useful for future research.

## Introduction

Kidney disease is increasingly concerned in the public health problems due to its growing prevalence and high cost of treatment. It refers to various injuries and diseases that affect the structure or function of the kidney, which is manifested by impaired kidney function, elevated blood pressure, abnormal urine and other symptoms. The etiology of kidney diseases is very complex, including congenital factors, genetic factors, environmental factors and living habits and other aspects. The molecular mechanisms of kidney disease have long been explored, mainly includes inflammation, apoptosis, oxidative stress, calcium overload, immune complex deposition, complement activation, mitochondrial dysfunction and so on [1–3]. Recently, with the continuous progress of science and technology, new research results are also emerging.

Annexins are a well-known multigene and multifunctional family of peripheral membrane-binding proteins [4]. In mammals, annexins are divided into annexin A1–A11 and A13 [5]. As an evolutionarily ancient and conserved family, Annexins share a highly homologous core domain, which includes an N-terminal domain and four alpha helical repeats with potential Ca^2+^-binding activity, and can bind phospholipids in a Ca^2+^-dependent manner [6]. Anxa2 is among the most extensively investigated annexin, especially with respect to mammalian biology [7,8]and human disease [9,10]. The biological functions of Anxa2 are associated with membrane trafficking [11], vascular homeostasis [12], signal transduction [13] and DNA integrity [14]. In addition, Anxa2 is involved in cell proliferation, survival, invasion and metastasis. Anxa2 facilitates fibrinolysis, the activation of inflammation and the immune system, and tissue damage and repair. As a result, Anxa2 dysfunction is associated with a variety of human diseases. However, few papers have focused on reviewing the research on Anxa2 in the kidney. Here, we will summarize the role of Anxa2 in renal physiological and pathological conditions.

## Characteristics of Anxa2

**Figure 1.**
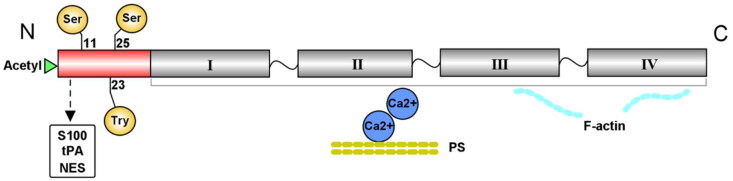
Structure of the Anxa2 protein. Anxa2 contains a highly divergent N-terminus and a conserved core domain in the C-terminus. The variable N-terminus includes one acetylation site and the three main phosphorylation residues Tyr23, Ser11 and Ser25. This N-terminal region also contains a binding site for the S100 protein family, tPA and NES. The Anxa2 core domain in the C-terminus consists of four homologous repeats. This domain has the ability to bind phospholipids in a Ca^2+^-dependent manner and interact with F-actin. tPA = tissue plasminogen activator, NES = nuclear export signal.

The human Anxa2 protein is the product of the *Anxa2* gene, and *Anxa2* is composed of 16 exons on chromosome 15q22. It is a 37 kDa curve-shaped protein with concave and convex sides that is distributed in human epithelial cells, endothelial cells, trophoblasts, tumor cells, and innate immune cells [6]. Anxa2 consists of two main structural domains, as depicted in Figure 1.

The head domain of Anxa2 contains a highly divergent N-terminus that is unique from other annexins [15]. Multiple functionally important sites have been identified in the variable N-terminus, including one putative acetylation site and the three main phosphorylation residues Tyr23, Ser11 and Ser25 [16]. These three residues can be phosphorylated by Src family tyrosine kinases and serine kinases, and are related to ligand interactions and the regulation of Anxa2 functions [16–18]. In addition, the first 12 N-terminal amino acids of this domain act as a binding site for the S100 protein family, which serves as the ligand of Anxa2 [19,20]. The main ligand of Anxa2 in the S100 family is S100A10 (also named p11) [21]. Generally, Anxa2 exists as a free monomer. When two molecules of Anxa2 are bound to a S100A10 homodimer, they form a heterotetrameric complex [22]. The Anxa2-S100A10 complex is preferentially enriched at the plasma membrane as an extracellular receptor or intercellular transporter [23]. This region also includes a binding site for tissue plasminogen activator (tPA) at positions 7–12 [24]. The binding of Anxa2 and tPA on the surface of endothelial cells has been extensively implicated in plasminogen activation and cell adhesion. This interaction can promote neoangiogenesis and the clearance of thrombi, thus maintaining vascular homeostasis [25,26]. Furthermore, monomeric Anxa2 can bind to a nuclear export signal (NES) through residues 3–13 of its N-terminal region [27]. Nuclear Anxa2 has been reported to be involved in DNA synthesis, mRNA processing or interactions with other nuclear factors [28,29]. In addition, nuclear translocation of Anxa2 protects cells against DNA damage and oxidative stress [30].

Similar to other annexins, Anxa2 has a highly conserved core domain in the C-terminus, which contains four homologous repeats of 70 amino acid residues. Each repeat consists of five α-helices [31]. The Anxa2 core domain has the ability to bind negatively charged lipophilic membranes in a Ca^2+^-dependent manner [4]. There is accumulating evidence that the Anxa2 core domain also interacts with F-actin within cells [32]. Accordingly, Anxa2 is considered a membrane-cytoskeleton linking protein that simultaneously binds to phospholipids and F-actin [33]. In addition, the Anxa2 core domain contains the binding site for plasminogen and heparin [34].

Overall, due to its specific molecular structure, Anxa2 performs various biological roles in human cells, including regulating membrane trafficking, acting as the main interactor in plasmin production and mediating signal transduction [7, 35].

## General functions of Anxa2

### Anxa2 in immune system

Anxa2 plays multiple roles in different stages of inflammation and may lead to various human disease states associated with acute and chronic inflammation [36]. As reported, when the cell is injured, Anxa2 increases on the cell surface and is involved in innate and adaptive immune responses through binding to pathogen proteins, C1q, TLR, anti-dsDNA antibodies and immunoglobulins [37]. Additionally, when a pathogen or insult is introduced, Anxa2 maintains vascular integrity, thereby preventing edema and the extravasation of blood cells [38]. once vascular integrity is lost, some inflammatory leukocytes, such as monocytes and neutrophils, can be recruited to a site of injury *via* Anxa2-mediated mechanisms [39,40]. Additionally, Anxa2 triggers different pathways within the multifunctional inflammasome system in varying inflammatory contexts. Downregulating Anxa2 impairs NLR family CARD-containing protein 4 inflammasome (NLRC4) activation by pattern-recognition receptors (PRRs), leading to defective caspase-1 activation and decreased interleukin-1β (IL-1β) and IL-18 secretion [41]. However, Anxa2 is involved in endolysosomal membrane repair and blocks NLRP3 inflammasome activation [42]. Anxa2 is also a regulator of endoplasmic reticulum stress, reducing unfolded protein response by upregulating the inositol-requiring enzyme 1 (IRE1) - (X-box binding protein 1) XBP1 pathway [43]. In addition, Anxa2 deficiency causes immune dysregulation and end-organ injury in human autoimmune disorders, such as atypical hemolytic uremic syndrome and antiphospholipid syndrome [44]. Overall, these results indicate that Anxa2 is a key regulator of inflammatory responses.

### Anxa2 in endothelium function

As an important cell surface fibrinolytic receptor, the heterotetrameric Anxa2 complex binds tPA and plasminogen, promoting the production of plasmin on the surface of endothelial cells and maintaining hemostatic and vascular homeostasis [45]. Anxa2^-/-^ mice have two to three times more fibrin accumulation in organs such as the kidneys, lungs, and heart than wild-type mice [26]. Anxa2 also facilitates the secretion of hemostatic factors by vascular endothelial cells, such as von Willebrand factor (vWF) [46]. However, overexpression of Anxa2 causes hemorrhage in acute promyelocytic leukemia (APL), and its impairment or decrease leads to thrombosis [47]. Furthermore, studies indicate that recombinant Anxa2 may be an antithrombotic therapy for humans. In a mouse model of oxygen-induced retinopathy, Anxa2 participates in the formation of retinal neovascularization *via* the PI3K/AKT axis [48]. Meanwhile, Anxa2 promotes the development of brain vessels by activating AKT/ERK signals [49].

### Anxa2 in tissue repair and fibrosis

Anxa2 is central to wound healing and promotes angiogenesis during tissue injury caused by oxidative damage, hypoxia and chronic tissue inflammation. During the sarcolemmal repair process, Anxa2 interacts with dysferlin to promote actin accumulation at the site of injury [50]. Furthermore, the injury-induced increase in cytosolic Ca^2+^ facilitates the fusion of Anxa2 and dysferlin-rich vesicles to repair damaged membranes [51]. Anxa2 also promotes cell detachment and migration by enhancing the endocytosis of β1-integrin for wound closure. In addition, Anxa2 may directly or indirectly participate in fibrogenesis in various organs when the injury-repair response is dysregulated, such as pulmonary fibrosis [14], cardiac fibrosis [52], hepatic fibrosis [53,54]. Overall, these studies show that Anxa2 plays a significant role in the physiologic response to tissue repair and fibrosis.

In addition to the functions described above, increasing evidence suggests that Anxa2 regulates tumor growth and progression. The mechanism mainly involves the overproduction of plasmin on the surface of endothelial cells and/or invasive cancer cells, as well as the recruitment of proangiogenic inflammatory cells into the tumor microenvironment [55]. Anxa2 overexpression has been observed in renal cell carcinoma [56], colorectal cancer [57], lung cancer [58] and hepatocellular carcinoma [59]. In addition, the involvement of Anxa2 in angiogenesis has been highlighted in many malignancies, including aggressive breast cancer [60] and glioblastoma [61]. Thus, abnormal Anxa2 expression may serve as a cancer diagnostic biomarker, predictive factor, and therapeutic target.

In summary, Anxa2 is involved in not only cell-surface fibrinolysis but also various cellular functions, including the inflammatory response, injury signaling, wound healing, and tumor progression, thus indicating its involvement in various human pathologies. We will present a map of Anxa2 to help us better understand its role in the kidney.

### Physiological role of Anxa2 in the kidney

Anxa2 is expressed in the normal renal cortex and medulla in mammals [62]. Under different physiological conditions, the forms and locations of Anxa2 are diverse. In a low-calcium environment relative to the renal non-damaged state, Anxa2 is expressed mainly in the cytoplasm in its soluble form. Extracellular membrane-bound Anxa2 is upregulated in response to physiologic stress and various injuries [63]. Moreover, Anxa2 is released locally in serum, urine and other body fluids because of its secretory properties [60, 64].

### Role of Anxa2 in vesicle-mediated transport

Recent studies have shown that Anxa2 is multifunctional in the kidney. First, Anxa2 is a major component of vesicle-mediated transport. Lipid raft microdomains generally serve as platforms for membrane organization. Anxa2 is a novel auxiliary factor for raft-containing vesicle routing from the Golgi apparatus to the apical membrane in polarized epithelial cells [65]. Channel proteins in the kidney help to maintain systemic water and salt homeostasis. There is accumulating evidence that Anxa2 is responsible for the fusion of channel proteins in renal epithelial cells [66]. For example, cAMP-elevating agents shift Anxa2 abundance from the cytosol to lipid rafts in the plasma membrane, which promotes cAMP-induced aquaporin-2 (AQP2) exocytosis [67]. Anxa2 is therefore required for the insertion of vesicles containing the water channel AQP2 into collecting duct renal cells. In addition, Anxa2 plays a crucial role in Na^+^-K^+^-2Cl^-^ cotransporter (NKCC2) trafficking in response to stimulation by vasopressin (AVP) or a low chloride state. Exocytosis of the ion channel NKCC2 into the apical membrane is followed by the phosphorylation of Anxa2 in the renal medulla [68]. Moreover, Anxa2 associates specifically with Ca^2+^‐selective channels, such as transient receptor potential vanilloid 5 (TRPV5) and TRPV6, in renal tubular epithelial cells. Anxa2 can mediate TRPV5 and TRPV6 translocation to the apical plasma membrane of polarized epithelial cells [69].

### Role of Anxa2 in cytoskeleton dynamics

Second, cytosolic Anxa2 harbors a C-terminal actin binding site and is linked to actin-cytoskeleton dynamics. In Madin-Darby canine kidney cells (MDCK) cells, the Anxa2-S100A10 complex at the plasma membrane can bind to the actin cytoskeleton, causing the association of cell–cell adhesion molecules (E-cadherin and nectin1) to form adherens junctions [70]. In addition, the small GTPase Rac1 can rapidly shift to the site of cadherin-mediated cell–cell contacts by interacting with Anxa2. These results indicate that Anxa2 is involved in the regulation of cell migration and cell–cell adhesion in epithelial cells [71].

### Role of Anxa2 in vascular homeostasis

Finally, Anxa2 acts as an extracellular membrane receptor for tPA on endothelial and multiple cancer cells, is involved in certain signal transduction events and has antithrombogenic properties [72,73]. The Anxa2-S100A10 complex can reorganize tPA and participate in the renal fibrinolysis system. The key function of Anxa2 is to maintain vascular integrity, which controls neutrophil chemotaxis. It helps regulate the immune response by enabling degradation of the extracellular matrix on the cell surface and inhibiting the chemotaxis of macrophage [74].

**Figure 2.**
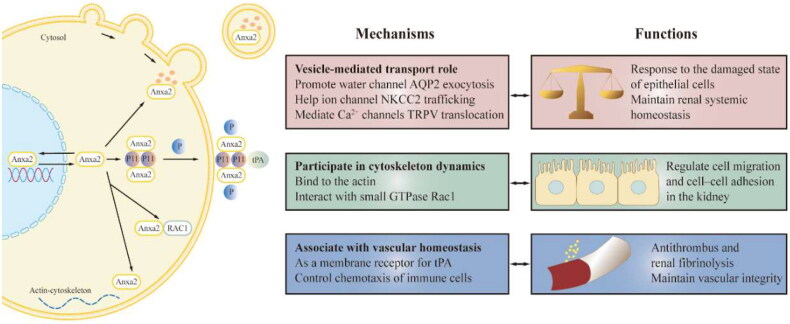
Physiological function of Anxa2 in renal cells. Anxa2 in the cytoplasm is linked with vesicles that mediate transport events (including water channel, ion channel, Ca^2+^‐channels) and the cytoskeleton dynamics. Extracellular membrane Anxa2 is a receptor for tPA, is involved in certain signal transduction pathways and has anti-thrombogenic properties and renal fibrinolysis. Anxa2 help control chemotaxis of immune cells to maintain vascular integrity. Anxa2 can also be released locally in serum, urine and other body fluids. tPA = tissue plasminogen activator, P11= S100A10, P = phosphorylation.

Importantly, Anxa2 appears to have several physiological roles in the kidney, as shown in Figure 2.

### Anxa2 in AKI

The most common pathophysiology of AKI is renal ischemia and acute tubular necrosis caused by nephrotoxic drugs, obstruction, sepsis or surgical operation. Others include acute interstitial nephritis, glomerular disease and vascular disease [75].The concentration of cytosolic calcium in proximal tubular epithelial cells is increased after acute kidney injury [76]. As major Ca^2+^-binding proteins, numerous studies have confirmed that Anxa2 can interact with S100 in a calcium-dependent manner *in vivo* and *in vitro* [77,78]. In renal ischemia–reperfusion injury (IRI), Anxa2 and S100A6 were immediately increased after the induction of acute tubular necrosis. Similarly, in toxin-mediated AKI, such as nephrotoxin uranyl nitrate and folic acid mouse models, Anxa2 and S100A6 were significantly increased. Further renal staining indicated that highly expressed Anxa2 and S100A6 colocalized with proliferating cell nuclear antigen (PCNA) in regenerating proximal tubular cells. After the recovery process, these proteins returned to normal levels. This study indicates that Anxa2 plays a critical role in regulating renal cell proliferation and regeneration [79,80].Meanwhile, in the renal tubulointerstitium after acute ischemia/reperfusion, large production of Anxa2 from postischemic kidneys could bind to factor H and prevent factor H from binding to the cell surface, thus triggering complement activation *via* the alternative pathway (AP). When factor H-deficient mice were injected with Anxa2, substantial and continuous deposition of complement 3b was observed in the renal tubule interstitium [81]. Anxa2/S100A10 was also reported to act as a pH sensor by interacting with cellular membranes in response to intracellular acidification during acute hypoxic conditions in human embryonic kidney cells [82]. Furthermore, in an acute polymicrobial sepsis mouse model, Anxa2 deficiency increased myeloperoxidase (MPO) level and bacterial loads in renal tissues resulting in more severe renal damage [83]. Additionally, in cisplatin-induced AKI mice, total expression of Anxa2 was upregulated in renal tubules. Further mechanistic analysis revealed that Anxa2 significantly induced the β-catenin/transcription factor EB (TFEB) signaling pathway to enhance lysosomal autophagy, ultimately alleviating AKI [84]. Therefore, increasing Anxa2 level can reduce AKI caused by multiple etiologies, and exogenous Anxa2 is expected to be a therapeutic agent for treating AKI.

### Anxa2 in CKD

The pathophysiology of CKD includes nephron loss, nephron hypertrophy, impaired glomerular filtration, and renal fibrosis [85]. Among them, renal fibrosis is the main pathological change and common pathway of a variety of chronic kidney diseases that eventually lead to renal failure. Renal fibrosis involves inflammatory response, apoptosis of innate cells and immune cells of kidney, enhancement of oxidative stress response, and imbalance of fibrosis-promoting/inhibiting cytokines [86]. Anxa2 may be an inflammatory regulator in CKD and play a pivotal role in the regulation of cell proliferation, activation, apoptosis, and coagulation by recruiting plasminogen and tissue plasminogen activator [87]. During the course of unilateral ureteral obstruction (UUO), tPA could trigger the clustering and interaction of Anxa2/CD11b, resulting in the subsequent activation of the integrin-linked kinase (ILK)-associated canonical nuclear factor-κB (NF-κB) pathway in macrophages [88]. In support of this finding, tPA could promote M2-to-M1 macrophage polarization through Anxa2-mediated NF-κB signaling events, leading to sustained interstitial macrophage accumulation and eventually irreversible chronic kidney fibrosis [89]. Jing Chen et al. used RNA sequencing to show that Anxa2 knockdown in proximal renal tubule cells affected multiple inflammatory signaling pathways, including interferon- and cytokine-mediated signals. Anxa2 knockdown upregulated the expression of chemokine ligand (CCL5) and IFN-related genes to regulate renal inflammation. CCL5 was found to recruit mononuclear cells to damaged proximal tubular cells, and dysregulation of CCL5 also leads to increase and activate inflammatory macrophages. In addition, Anxa2 regulates selective splicing of NF-κB genes, induces inflammatory responses and promotes the progression of CKD [90].

Compared with that in normal controls, Anxa2 was significantly increased and identified as a candidate target for autoantibodies in patients with proliferative lupus nephritis (pLN). Another novel finding in this study was that serum level of Anxa2 may be a specific injury index for pLN [91]. The binding of circulating Anxa2 with immunoglobulin in serum was correlated with the clinical and histological activity index in pLN patients [92]. Additionally, urine Anxa2 could act as a useful biomarker for progressive glomerular diseases [93]. The existence of vascular lesions in lupus nephritis (LN) biopsy samples seemed to be related to increased vascular expression of Anxa2 [94]. Regarding anti-renal autoreactivity, a positive anti-Anxa2 autoantibody test is associated with active LN [95]. Furthermore, Anxa2 was identified as the primary target of CD4^+^ T cells [96]. Increasing Anxa2 mediates the binding of anti-dsDNA antibodies to the mesangial cell surface and matrix, and anti-dsDNA antibodies are internalized into the cytoplasm and nucleus, resulting in nephritis and fibrosis associated with manifestations in LN [97].

In an analysis of kidney-specific networks between glomeruli and tubulointerstitium, Anxa2 was a core factor in regulating the pathogenesis of diabetic nephropathy (DN) [98]. Moreover, when recombinant Anxa2 protein (rAN II) was administered to a mouse model of 2-type diabetes for 8 weeks, kidney weight, albuminuria and the glomerular lesion region were less than those of the control. However, bleeding time, prothrombin time (PT), and active partial thromboplastin time (APTT) did not significantly differ between the two groups, suggesting rAN II may delay the progression of diabetic nephropathy without affecting the coagulation system [99]. In human renal glomerular endothelial cells, glomerular DNA damage induced collagen type VI (COL6) secretion and deposition through the Anxa2-mediated pathway. Silencing Anxa2 with siRNA could inhibit mitomycin C-induced COL6 secretion and alleviate nodular glomerular sclerosis [100]. These data suggest that Anxa2 participates in the progression of DN.

The expression and function of Anxa2 have also been reported in other types of CKD. For example, the Anxa2/S100A10 complex is an endogenous binding partner of phospholipase A2 receptor (PLA2R) at the podocyte cell surface that regulates PLA2R function in membranous nephropathy (MN). In addition, extracellular vesicles contained Anxa2/S100A10-PLA2R, which could be quantitated as a target in MN [101]. Anxa2 is highly increased in mesangial proliferative glomerulonephritis (MePGN) [102]. Significant expression of Anxa2 autoantibodies was also detected in children with primary nephrotic syndrome (PNS). In animal studies, Anxa2 antibodies were shown to contribute to Rho signals by reducing its binding to protein tyrosine phosphatase, resulting in cytoskeletal redistribution and injury in podocytes and eventually leading to high level of albuminuria [103].

### Anxa2 in renal cell cancer

As discussed previously, an increase in Anxa2 was correlated with adverse and severe outcomes of tumors [36, 104]. This was consistent with the overproduction of Anxa2 protein in clear-cell renal cell carcinoma (RCC) and represents invasive and metastatic potential [56, 105,106]. Furthermore, strong Anxa2 expression in the kidney correlated with RCC differentiation. therefore, Anxa2 was a useful prognostic indicator [107,108]. Anxa2 was significantly increased in the urine of patients with upper tract urothelial carcinoma (UTUC) compared with healthy individuals [109]. Due to the local release of Anxa2 in the human body, soluble Anxa2 has also been detected in the renal venous blood of some patients with RCC [64]. These findings indicate that Anxa2 in the urine or serum is an effective marker for the clinical prediction of renal cell cancer.

The molecular mechanism by which Anxa2 participates in RCC involves regulating the cytoskeletal remodeling of actin and promoting cancer cell motility [110]. In a ferric nitrilotriacetate (Fe-NTA)-induced rat RCC model, renal proximal tubules were damaged by oxidative stress. High level of Anxa2 were detected and phosphorylated at serine and tyrosine residues, suggesting that Anxa2 regulation of kinase systems was involved in RCC [111]. Anxa2 and S100A10 also form a complex on the RCC cell surface and activate plasmin generation and extracellular matrix deposition [112]. Anxa2 also interacts with dihydrolipoamide branched chain transacylase E2 (DBT) to initiate the Hippo pathway, resulting in decreased nuclear localization of yes1-associated transcriptional regulators (YAP) and the alleviation of lipid accumulation, ultimately inhibiting the progression of clear cell RCC [113]. Moreover, hematopoietic stem cells in Anxa2-deficient mice express reduced level of CXC-chemokine receptor (CXCR4) [114]. The CXC-chemokine ligand CXCL12/CXCR4 biological axis is a major determinant of RCC metastasis [115]. These studies suggest that Anxa2 might promote RCC metastatic and invasion potential by regulating the CXCL12/CXCR4 biological axis. Another mechanistic study revealed that adding purified Anxa2 to lymphocyte cultures inhibited cell proliferation, suggesting that an increase in soluble Anxa2 was immunosuppressive [64]. The use of Anxa2 antibodies has also been reported in other cancer treatments. For example, using anti-Anxa2 antibodies to treat human breast cancer cells proved effective in preventing cancer development [116]. Anxa2 siRNA could inhibit prostate cancer growth without detectable adverse effects [117].These strategies might be the focus for further investigations on pharmaceutical intervention in renal cell cancer.

### Anxa2 in nephrolithiasis

Evidence to date suggests that most kidney stones are primarily composed of calcium oxalate monohydrate (COM) [118]. Anxa2 is known as a calcium binding protein that can help COM crystal adhesion *via* the crystal structure [119,120]. Kumar et al. suggested that Anxa2 could mediate the rapid adhesion of COM crystals to the renal epithelial cell surface [121]. Confocal microscopy confirmed the colocalization of Anxa2 and Caveolin-1, which is involved in endocytosis and exocytosis, on the apical membrane of Madin-Daby canine kidney cells [122]. These results provided evidence that Anxa2 could not only avidly bind to COM crystals but could also participate in subsequent stone internalization. Pretreatment with antibodies against Anxa2 dramatically decreased COM crystal adhesion compared to that in the control. However, the anti-Anxa2 antibody did not completely eliminate COM crystal binding, suggesting that Anxa2 may be one of many crystal binding molecules on the renal epithelial cell surface [121]. The disruption of chloride (Cl^-^) channel-5 (CCL5) resulted in the translocation of Anxa2 from the cytoplasm to the luminal cell surface, which was accompanied by increased level of crystal binding that could be blocked with Anxa2 antibodies. The role of Anxa2 in crystal binding was supported in an experimental cell culture model of Dent’s disease [123]. In the future, blocking COM crystal adhesion with antibodies against Anxa2 or other binding molecules may alleviate tubular damage caused by tubular calcinosis.

## Conclusion

In summary, this review provides an overview of the role of Anxa2 in physiological and pathological renal conditions. Under different states, there are diverse forms and locations of Anxa2. Originally, Anxa2 is a crucial phosphorylated and calcium binding protein encoded by a nuclear gene and transported to the cytoplasm. Cytosolic Anxa2 can mediate ion and water channel proteins or unknown vesicles trafficking to the plasma membrane in renal epithelial cells. Additionally, Anxa2 binds to p11 to form a heterotetrameric complex at the cell surface and act as a functional receptor. Furthermore, Anxa2 not only stabilizes the cytoskeleton but can also be shed into urine and serum. However, the function of Anxa2 in different kidney diseases is varied and intricate. In the early stage of renal tubular injury, the increase in Anxa2 expression is related to cell proliferation and regeneration and triggers renal complement activation. A continuous increase in Anxa2 level promotes inflammatory responses and initiates damage binding to auto-antibodies in renal diseases. Of note, Anxa2 can be an injury index to predict the prognosis of chronic glomerular diseases or renal cancer. Anxa2 is also associated with calcium-related nephrolithiasis.

**Figure 3.**
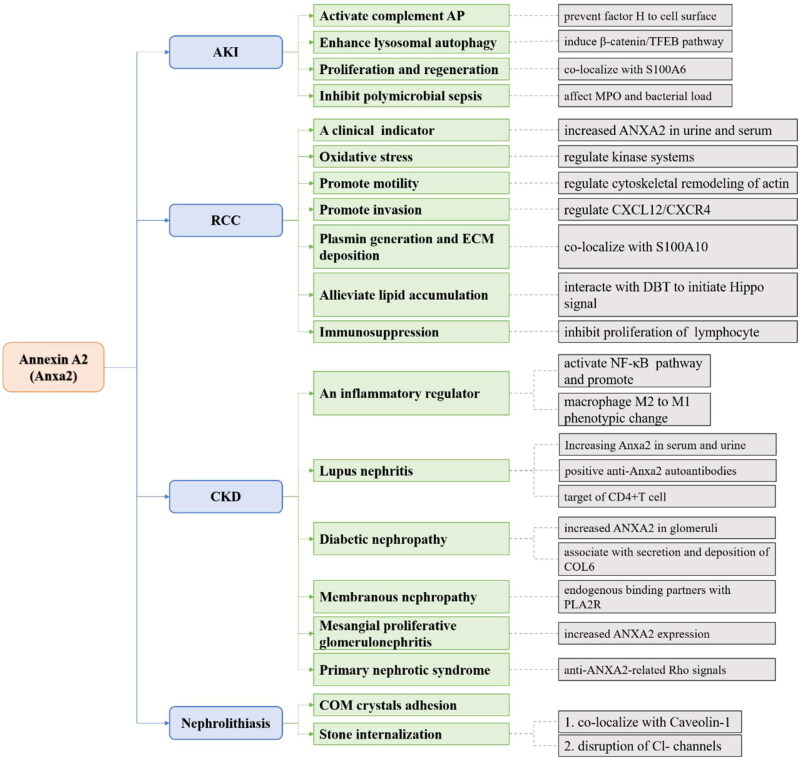
Overall graph showing the Anxa2 signaling pathway in different kidney diseases (including AKI, CKD, RCC and nephrolithiasis).

We provide some mechanistic insight into Anxa2 in the kidney in this review (Figure 3). There is a growing body of evidence focused on Anxa2 in renal diseases. Further studies are needed to determine its function and develop effective therapeutic methods to treat renal diseases by directly targeting Anxa2.
